# The Esthetic Use of Botulinum Toxins in Cancer Patients: Providing a Foundation for Future Indications

**DOI:** 10.3390/toxins17010031

**Published:** 2025-01-10

**Authors:** Marco Papagni, Monica Renga, Selene Mogavero, Paolo Veronesi, Maurizio Cavallini

**Affiliations:** 1 Executive Committee of Agorà—Italian Society of Aesthetic Medicine, 20122 Milan, Italy; monica.renga@gmail.com (M.R.); maurizio.cavallini@libero.it (M.C.); 2 Scientific Writing, 06089 Torgiano, Italy; selene.mogavero@scientificwriting.it; 3 Division of Breast Surgery, European Institute of Oncology, Istituto di Ricovero e Cura a Carattere Scientifico IRCCS, 20141 Milan, Italy; paolo.veronesi@ieo.it; 4 Department of Oncology and Hematology, University of Milano, 20122 Milan, Italy

**Keywords:** botulinum toxins, BoNTs, oncology patients, esthetic medicine, safety, QoL

## Abstract

Advances in oncological treatments have improved the survival rates of cancer patients but have often resulted in significant physical changes that negatively impact their self-esteem and psychological well-being. Cancer patients frequently ask esthetic practitioners to perform procedures to address such changes. However, practitioners often hesitate to satisfy such requests due to lacking guidelines or recommendations. The use of botulinum toxins (BoNTs) for esthetic purposes has shown significant promise in improving the quality of life for cancer patients. This review explores the broad application of BoNTs in many medical branches, focusing on oncology. A substantial amount of literature shows that BoNTs are safe and effective as a type of adjunctive therapy compared to classical cancer treatments. We provide our expert opinion that the use of BoNTs for esthetic purposes is safe for cancer patients and even recommended for those whose mood is influenced by the worsening of their physical appearance. Careful patient selection and interdisciplinary collaboration are essential to the safe integration of BoNTs into cancer care.

## 1. Introduction

In our clinical practice, we are increasingly encountering requests for esthetic therapies from patients who have experienced significant changes in their appearance due to illness, life-saving drug treatments, or trauma. These changes have negatively impacted their self-esteem and quality of life (QoL). A substantial proportion of these patients are those diagnosed with cancer and undergoing treatment. Thanks to advancements in early diagnostics and therapies, many cancer patients now have a much longer life expectancy and require treatments to manage the disease over extended periods [1]. However, some life-saving treatments can exacerbate skin deterioration and aging, causing additional distress, particularly in younger patients [2,3,4]. For many, the diagnosis of cancer and subsequent treatments cause profound shock, altering people’s daily lives, mood, and overall well-being [5]. The changes to their body and self-image can significantly worsen their psychological state during an already challenging period [6].

It is well established that maintaining a positive mood and psychological state is crucial in the therapeutic recovery pathway for any disease, particularly in oncology, where the immune system, which can be influenced by psychological well-being, plays a critical role [7]. Oncologists often encourage their patients to maintain a lifestyle as normal as possible and not to allow the disease to define their lives while being mindful of treatment-related contraindications. The approach to cancer patient care is increasingly holistic, extending beyond purely pharmacological treatments targeting cancer cells to encompass broader considerations of the patient’s mood and QoL [8].

For certain cancer patients, a multidisciplinary team of resources and professionals is now available to support the oncologist in managing the patient’s QoL during treatment. This comprehensive care is essential to addressing the disease’s clinical aspects and personal consequences [9]. However, the guiding principle for any adjunctive therapeutic intervention must always be the safety of the patient in an altered physiological state [10].

In recent years, we, as practitioners in the medical esthetic field, have increasingly received requests for assistance from cancer patients. However, when considering their options according to science and conscience, practitioners often cannot rely on adequate scientific bibliographic references to provide guidelines or recommendations. Without the appropriate scientific support to guarantee a safe and proper response to these patients, we usually cannot fulfill these requests despite having the necessary tools.

When approaching these patients in the medical esthetic setting, it is crucial to consider two safety-related aspects: first, any therapeutic proposals should not adversely affect the primary pathology or its treatment, and second, the underlying pathology or therapies should not negatively impact the proposed esthetic procedure or expose the patient to additional risks. There is insufficient scientific support to guide us in making these choices.

Biostimulators are generally discouraged for oncologic patients undergoing treatment, particularly during the phases of prevention or cytoreduction before surgery. This recommendation is based on the fact that biostimulators can stimulate cell proliferation, which may not be suitable for patients with active cancer [11]. Furthermore, extra care should be taken when considering the use of fillers during cancer treatments due to the immunocompromised state of these patients. The inflammatory condition of the skin, exacerbated by cancer treatments, poses a significant risk for infections and inflammatory reactions at the site of filler application [12,13,14].

Botulinum neurotoxins (BoNTs), a drug with numerous indications, are successfully and safely used across multiple medical specialties today [15]. Their use has efficiently treated neuropathic pain since its injection near the involved nerve endings can block pain neurotransmitters in the peripheral and central sensory systems [16,17]. They have also been employed in relieving symptoms of intractable head pain after lateral skull base surgery [18].

For patients experiencing gastroparesis, intrapyloric BoNT injection has been proven to be safe and can provide transient relief in children [19]. However, conflicting results call for revisiting recommendations to treat gastroparesis [20,21].

After stroke, BoNTs were employed to treat limb spasticity, improving muscle tone and disability assessment in the upper and lower limbs [22,23]. The treatment has transient effects, which is why it should be continuous. Indeed, patients who discontinued it because of the COVID-19 pandemic have reported worsening symptoms [24].

Additionally, BoNTs have been successfully employed in the gynecological field to treat conditions, such as chronic and myofascial pelvic pain, vestibulodynia, vaginism, dyspareunia, vulvodynia, overactive bladder, and urinary incontinence [25,26], and, similarly, it has been used in men to treat overactive bladder [27].

Relaxing glabellar muscles with BoNT injections may also help with depression. This may be explained by the facial feedback hypothesis, which states that facially expressed emotions can generate a feedback signal that maintains and reinforces the same feelings. Therefore, blocking a specific facial expression by means of BoNTs can interrupt the feedback mechanism and help with depression and comorbid migraine [28,29,30].

Currently, there are no established guidelines concerning the esthetic use of BoNTs in oncological patients. This review aims to lay the groundwork for initiating a meaningful dialogue on this important yet underexplored topic. By examining the safety and potential benefits of such interventions, we hope to open the door to further research and, eventually, the creation of formal guidelines. Our objective is to highlight that the esthetic use of BoNTs can be both safe and valuable for cancer patients when conducted carefully considering their unique medical context.

We will review the international scientific literature to identify adjuvant indications in the oncological setting that demonstrate BoNTs’ safe use, clinically and esthetically, to support emotional well-being. This includes researching any contraindications (even hypothetical, based on rationale) related to oncology and its therapies.

The work will also involve identifying any existing publications on using BoNTs for esthetic purposes in cancer patients and any literature studies on the positive psychological effects of toxin therapy in the upper third of the face.

Determining which types of patients and therapeutic situations present no contraindications to esthetic therapy with BoNTs is crucial. The aim is to provide esthetic and emotional support that could enhance acceptance and resilience to the underlying pathology and its treatment, positively impacting the patient’s QoL and, potentially, their prognosis.

Moreover, it is essential to consider that many patients who undergo cosmetic procedures before the onset of their disease and therapy are often forced to stop, which can feel like another defeat. If feasible, why not continue to support them?

## 2. Efficacy and Safety of Botulinum Toxins for Esthetic Use

BoNT injection is a highly effective and safe procedure in esthetic medicine, particularly in improving expressive facial wrinkles, including glabellar lines, crow’s feet, and forehead wrinkles. Numerous studies have repeatedly demonstrated its efficacy in providing temporary but significant improvements in the appearance of these lines, leading to enhanced patient satisfaction and self-esteem [12,31,32,33]. The safety profile of BoNTs is well documented, with adverse effects being generally mild and transient, such as localized pain, swelling, and transient muscle weakness [34,35].

However, applying BoNTs in esthetic medicine can be challenging [36,37,38]. The precision of injection techniques and dosage is crucial in order to avoid complications like eyelid ptosis or asymmetry [39]. Additionally, patient selection is essential, as individuals with certain neurological conditions or those on aminoglycosides may be at higher risk for developing adverse reactions [39,40,41]. There is also evidence of retrograde dissemination affecting the CNS after the peripheral injection of the toxin [42]. Despite these considerations, when administered by experienced practitioners, BoNT injection remains a highly effective and safe option for facial rejuvenation [40,43].

The esthetic use of BoNTs is not limited to enhancing expressive wrinkles in the upper third of the face [44]. Other successful applications include the following: off-label cosmetic injections in the neck and middle and lower areas of the face, including reduction in masseter muscle and platysma muscle [45]; the treatment of multiple eccrine hidrocystomas, which has proven simple and safe, with no scarring [46,47,48]; the prevention of widening of facial scars during revision surgery [49]; the treatment of keloids by means of intralesional injection [50]; restoring facial symmetry after permanent facial paralysis [51]; correcting capsular contracture after reconstructive breast surgery [52]; treating buccinator in patients with facial synkinesis [53]; injection concomitant to fat grafting for contouring of the forehead [54]; treating hyperhidrosis by targeting the parasympathetic cholinergic neurons [55]; treating gingival smile [56]; and treating bruxism by injecting the masseter muscle [57]. For subjects who prefer a more natural appearance, microbotox is a valid alternative if they want to avoid the frozen-face effect of BoNTs. It consists of intradermal injections of BoNT microdroplets, and it has been successfully employed for facial rejuvenation; mid-lower face-lifting; and fine wrinkle reduction in the forehead, periocular, and cheek regions [58].

## 3. Botulinum Toxins: Non-Esthetic Use in Cancer Patients

Numerous articles exist on the adjuvant uses of BoNTs in oncology. These studies highlight various scenarios where the toxin is effective, including by increasing the uptake of chemo- and radiotherapy by means of the neoplastic mass. Table 1 summarizes a selection of studies documenting the use of BoNTs as (adjuvant) therapy in cancer patients.

Additionally, we acknowledge a few published reviews that nicely summarize the available literature on the use of BoNTs in the broad area of oncology.

Shaw and colleagues reviewed the use of BoNTs as an intervention for side-effect management and the prevention of complications in patients who undergo surgery for head and neck cancers and esophageal cancer. Overall, positive effects were found for hypersalivation or other salivation issues, esophageal stricture, and the management of gastric emptying [59].

Mittal and Jabbari focused on the use of BoNTs in post-radiation and post-surgical cancer pain, its repairing or healing function after radiation- or surgery-damaged parotid glands, and how BoNTs affect cell growth and apoptosis in cancer cell lines. They conclude that the use of BoNTs in these scenarios is promising: the local injection of BoNTs relieves neuropathic pain and local muscle spasms near the site of radiation or surgery; similarly, BoNT injection has good efficacy in treating gustatory hyperhidrosis or managing post-parotidectomy fistula and sialocele; finally, the effects of BoNTs on several cell lines were positive. However, the authors also warned that more blinded and placebo-controlled studies are required to verify these encouraging results [60].

In their mini-review, Grenda et al. reviewed the use of BoNTs in cancer therapy and came up with three major conclusions. First, they agreed that BoNTs could be a valid tool in cancer treatment; the effect could be analgesic after cancer radiotherapy or it could directly inhibit tumor growth and promote the apoptosis of cancer cells [61].

In their systematic review, Safarpour and Jabbari carefully identified four primary ways of effectively using BoNTs: the reduction in pain after local injection at the site of surgery or radiation; the prevention of gastroparesis after the local resection of esophageal cancer by injection into the pylorus; the reduction in facial swelling during eating after injection the parotid region of patients who underwent parotid gland surgery; and the cessation of severe jaw pain after the first bite (first-bite syndrome, complications of parotidectomy, etc.) if BoNTs were injected into masseter muscle [62].

Although BoNTs are safe to use in many oncological conditions, there are cases where its use requires careful consideration due to potential contraindications and immunological responses. Specifically, the use of BoNTs is contraindicated in patients with thymoma and other tumors where myasthenia is present as a paraneoplastic syndrome. For instance, BoNTs should be avoided in patients with Lambert–Eaton myasthenic syndrome (LEMS). This neuromuscular autoimmune disorder targets junctions and is characterized by weakness, autonomic dysfunction, and areflexia, frequently correlated to small-cell lung cancer [63,64]. BoNTs should be avoided primarily due to the risk of exacerbating the neuromuscular symptoms associated with myasthenia, which can be worsened by the effects of botulinum toxins on neuromuscular transmission [65,66].

Moreover, in some lymphomas, there is a concern that immunomodulatory therapies could stimulate the immune system to produce antibodies against the botulinum toxin. While the presence of these antibodies does not directly threaten patient health, it can lead to a phenomenon known as immunoresistance, where the therapeutic effects of the toxin are diminished or lost altogether [67].

**Table 1:** Studies documenting the use of botulinum toxins as (adjuvant) therapy in cancer patients.

Reference	Type of Cancer/Condition Related to Cancer	Therapy	No. of Patients	AEs	Efficacy of BoNTs	Proposed Mode of Action
Andersen 2016 [68]	Gastric cancer	Intragastric injection of BoNTs.	6	None	One patient had no tumor growth between weeks 8 and 20 after treatment. One patient had a significantly improved clinical condition.	Blocking vagal nerve signals to suppress tumor growth.
Bhutani 2019 [69]	Gastroparesis after esophagectomy for distal esophageal cancer	A linear echoendoscope guided an aspiration fine needle into the pyloric sphincter, injecting 100 U of BoNTs into four quadrants. Then, a pyloric balloon dilated the pylorus to 20 mm.	1	None	Significant response to treatment, which lasted 6 months.	-
Bhutani 2022 [70]	DGE	Endoscopic intrapyloric BoNT injection with pyloric balloon dilation.	21	None	Very beneficial, leading to significant symptomatic improvements.	Reduction in pyloric motor activity and the acceleration of gastric emptying in patients with DGE.
Coarfa 2018 [71]	Prostate cancer	Patients were injected (ultrasound-guided, transrectal) with 100 U of BoNTs before prostatectomy 4 weeks later.	4	None	Denervation with BoNTs resulted in increased apoptosis in human prostate cancer.	Nerve inhibition changes the energetic metabolism of cancer cells and increases apoptosis.
Kim 2022 [72]	Pain after muscle invasion of cancer	Ultrasound-guided BoNT injection to the psoas muscle.	2	None	BoNTs relieved the intractable pain for at least 9−12 weeks.	The inhibition of pain neurotransmitters at peripheral and central levels.
Lee 2017 [73]	DGE after pylorus-preserving gastrectomy in early gastric cancer patients	Four injections of 25−50 U BoNTs into each of the four quadrants of the prepyloric area.	6	None	All BoNT injections were successful.	BoNT interferes with the tonic contraction of the pylorus.
Marchese 2022 [74]	Pharyngocutaneous fistula after salvage surgery in head and neck cancer patients	Intraparotid injection of BoNTs.	13	None	BoNTs promoted the closure of the fistula. It decreased the subjective perception of the saliva flow.	-
Mittal 2012 [75]	Focal cancer pain after surgery or radiation	BoNTs (20–100 U) were injected into the focal pain areas (skin, muscle, or both).	7	Weakness of jaw muscles after bilateral masseter injection in one patient; this was not observed during the second injection (reduced dose)	Significant improvement in pain.	Inhibiting the release of pain transmitters, glutamate, substance P, calcitonin gene-related peptide, and proinflammatory agents (like prostaglandins, bradykinin, histamine, and cyclooxygenase from the nerve endings and sensory ganglia).
Mueller 2022 [76]	Parotid and submandibular gland radioprotection	The parotid gland received six injection points, and the submandibular gland received three injection points under ultrasound control, up to 30 U of BoNTs per injection point.	10	Only minor	Clinically relevant radioligand uptake reduction by the salivary gland.	Fully reversible glandular denervation, resulting in reduced radiation sensitivity.
Nam 2017 [77]	Neuropathic pain in a patient with a brain tumor	Subcutaneous injection of BoNTs (100 U) in the most painful area in the posterior left thigh.	1	None	Pain significantly decreased after the injection.	The inhibition of neurogenic inflammation and peripheral sensitization by attenuating the release of neuropeptides from nociceptive sensory neurons via peripheral SNAP-25 cleavage. BoNTs may also decrease the delivery to the neuron cell membrane of the transient receptor potential vanilloid-1, which intensifies the excitability of nociceptors.
Nevins 2020 [78]	DGE following esophagogastrostomy for cancer	BoNTs (100 U in 4 mL) were administered in a quadrantic fashion around the pylorus via endoscopy.	29	None	Successful management of DGE.	-
O’Donnell 2011 [79]	Pectoral muscle spasms after mastectomy	Two electromyography-guided injections of 100 U of BoNTs into the left pectoralis major muscle (25 U × four sites) or 250 U of Dysport into each pectoralis major muscle.		None	Complete resolution of symptoms.	-
Rostami 2016 [80]	Post-surgical and post-radiation pain in cancer patients	Injections of 100 U of BoNTs intramuscularly or subcutaneously.	12	None	Significant pain reduction in and improvement of VAS, PGIC, and QoL.	Blocking the release of several pain modulators from presynaptic vesicles in addition to acetylcholine.
Stevens 2018 [81]	TTF compliance in a patient with glioblastoma	Subcutaneous BoNT injections of the scalp and forehead every 3–4 months.	1	None	The hyperhidrosis and tolerance of TTF improved significantly.	-
Valenzuela 2019 [82]	Chemotherapy-induced first-bite syndrome in a patient with Hodgkin lymphoma	BoNT chemodenervation before his next chemotherapy session.	1	None	Reduction in pain by fifty percent two weeks later.	
Wong 2018 [83]	Strabismus and sixth nerve palsies caused by nasopharyngeal carcinomas.	BoNTs were injected at ~1 cm posterior to the insertion of the medial rectus muscle on the globe.	25	Transients ptosis	Earlier regaining of binocularity and reduction in diplopia.	Rectus muscle structural alteration produced by chemodenervation.

**Abbreviations: AEs**, adverse events; **BoNTs**, botulinum neurotoxins; **DGE**, delayed gastric emptying; **PGIC**, patient global impression of change; **QoL**, quality of life; **TTF**, tumor-treating field; **U**, unit; **VAS**, visual analog scale.

## 4. Cancer Patients, Quality of Life, Esthetic Procedures, and Botulinum Toxins

Cancer patients often experience significant changes in their QoL due to both the disease and its treatments, including physical alterations that impact their self-image and emotional well-being. Esthetic interventions have emerged as a supportive approach to improve the psychological state of these patients, addressing issues such as alopecia, facial asymmetry, scarring, and other skin conditions caused by chemotherapy or radiation.

Combining BoNTs and microneedling improved scar appearance following nonmelanoma skin cancer surgery [84]. Similarly, BoNT injection after surgery significantly improved the appearance of postoperative scars in a low-vs.-high dose comparison study [85]. One patient receiving treatment for chronic myeloid leukemia with imatinib mesylate developed blepharospasm. In their case report, the authors describe the successful use of BoNTs to treat the periorbital condition [86]. Also, in post-chemotherapy patients, hyaluronic acid with lidocaine in the nasolabial folds and BoNT injections to glabellar lines significantly improved wrinkle severity [87].

The role of these procedures extends beyond mere cosmetic enhancements; they serve as interventions to restore confidence and a sense of everyday life, which are crucial in boosting overall QoL and aiding emotional recovery.

The potential benefits of integrating esthetic procedures into the holistic care of cancer patients are well known. For example, addressing chemotherapy-induced alopecia or skin discoloration can reduce the visibility of the disease, help patients regain control over their appearance, and improve self-esteem and social reintegration [14]. However, these interventions must be carefully managed to ensure safety, particularly in patients with compromised health due to ongoing oncological treatments.

The potential use of BoNTs is promising due to its established efficacy in treating facial wrinkles and minimal invasiveness, making it a viable option even for patients with compromised health [14,87]. Addressing such cosmetic concerns can significantly improve patients’ mood and overall QoL, contributing to better psychological outcomes during and after cancer treatment.

Despite the small case series, a meta-analysis summarizing the results of five randomized controlled trials indicated that the enhancement of glabellar wrinkles with 29 to 50 U of onabotulinumtoxin A has a significant antidepressant effect without major side-effects [88].

Moreover, the psychological benefits of esthetic interventions should not be underestimated. As patients transition from active cancer treatment to survivorship, addressing the visible side-effects of therapy can provide closure and facilitate emotional healing. Surgical reconstruction and other cosmetic procedures have positively impacted this context, underscoring the importance of a multidisciplinary approach that includes esthetic care in oncology [89]. Interestingly, even patients who did not care much about their appearance found esthetic therapy helpful in improving their self-reflection and relating to others [90].

Esthetic procedures could offer valid options for managing side-effects, pain relief, and social interactions, improving the patient’s QoL, thus possibly leading to an enhanced adherence to the oncological treatment plans and increasing the chances of a favorable prognosis [91].

In particular, BoNTs, hyaluronic acid, and calcium hydroxyapatite were shown to be effective in improving asymmetries, volumizing surgical depressions, and dissembling atrophic scars in patients undergoing oculofacial prosthetic rehabilitation after the surgical treatment of head and neck cancer, enhancing their appearance and QoL [92].

In summary, incorporating esthetic interventions into cancer care can significantly improve patients’ QoL by focusing on physical and emotional needs, ultimately supporting better psychological outcomes and enhancing patient mood throughout their cancer treatment and healing journey.

Recently, the Cancer Association of South Africa (CANSA) published a paper, with the guidance of its physicians, describing the use of BoNTs in oncology. This paper promotes esthetic toxin procedures during therapeutic phases, noting that they can enhance patient mood and adherence to treatment plans by fostering a positive outlook (CANSA fact sheet) [93].

## 5. Conclusions and Recommendations

Using BoNTs as an esthetic intervention in oncology patients represents a promising approach that addresses the physical consequences of cancer therapy and the psychological impact of appearance changes. With its well-established safety profile, minimal invasiveness, and clinically proven efficacy, BoNTs stand out as being able to significantly improve the QoL of cancer patients who experience visible alterations due to their treatments.

Integrating BoNTs into oncological care requires collaboration between oncologists and esthetic practitioners to ensure a holistic, patient-centered approach. Therefore, establishing clear guidelines and therapeutic protocols for BoNT use in oncology patients could be crucial in advancing this field.

Based on our literature review and our personal clinical experience, sufficient evidence exists to rule out significant risks associated with the use of BoNTs in most cancer patients undergoing treatment.

Moreover, current research supports the notion that BoNTs can be safely used in a oncological context, even in patients undergoing active treatment, after the careful evaluation of blood tests, skin integrity, inflammation markers, and general medical history. The psychological benefits of esthetic procedures, such as improved self-image and mood enhancement, should not be underestimated, as these can help improve adherence to ongoing oncological therapies and an overall positive impact on patient well-being.

In conclusion, with careful patient selection and interdisciplinary collaboration, BoNT injection could become a standard adjunctive therapy in the holistic management of cancer patients, helping them navigate the emotional and esthetic challenges of their disease, treatment, and healing process.

## Data Availability

No new data were created or analyzed in this study. Data sharing is not applicable to this article.
